# Causative Links between Protein Aggregation and Oxidative Stress: A Review

**DOI:** 10.3390/ijms20163896

**Published:** 2019-08-09

**Authors:** Elise Lévy, Nadine El Banna, Dorothée Baïlle, Amélie Heneman-Masurel, Sandrine Truchet, Human Rezaei, Meng-Er Huang, Vincent Béringue, Davy Martin, Laurence Vernis

**Affiliations:** 1 Molecular Virology and Immunology Unit (VIM-UR892), INRA, Université Paris-Saclay, 78352 Jouy-en-Josas, France; 2 Institut Curie, PSL Research University, CNRS UMR3348, Université Paris-Sud, Université Paris-Saclay, 91400 Orsay, France

**Keywords:** protein aggregation, redox, oxidative stress, proteinopathy

## Abstract

Compelling evidence supports a tight link between oxidative stress and protein aggregation processes, which are noticeably involved in the development of proteinopathies, such as Alzheimer’s disease, Parkinson’s disease, and prion disease. The literature is tremendously rich in studies that establish a functional link between both processes, revealing that oxidative stress can be either causative, or consecutive, to protein aggregation. Because oxidative stress monitoring is highly challenging and may often lead to artefactual results, cutting-edge technical tools have been developed recently in the redox field, improving the ability to measure oxidative perturbations in biological systems. This review aims at providing an update of the previously known functional links between oxidative stress and protein aggregation, thereby revisiting the long-established relationship between both processes.

## 1. Introduction

**Figure 1:**
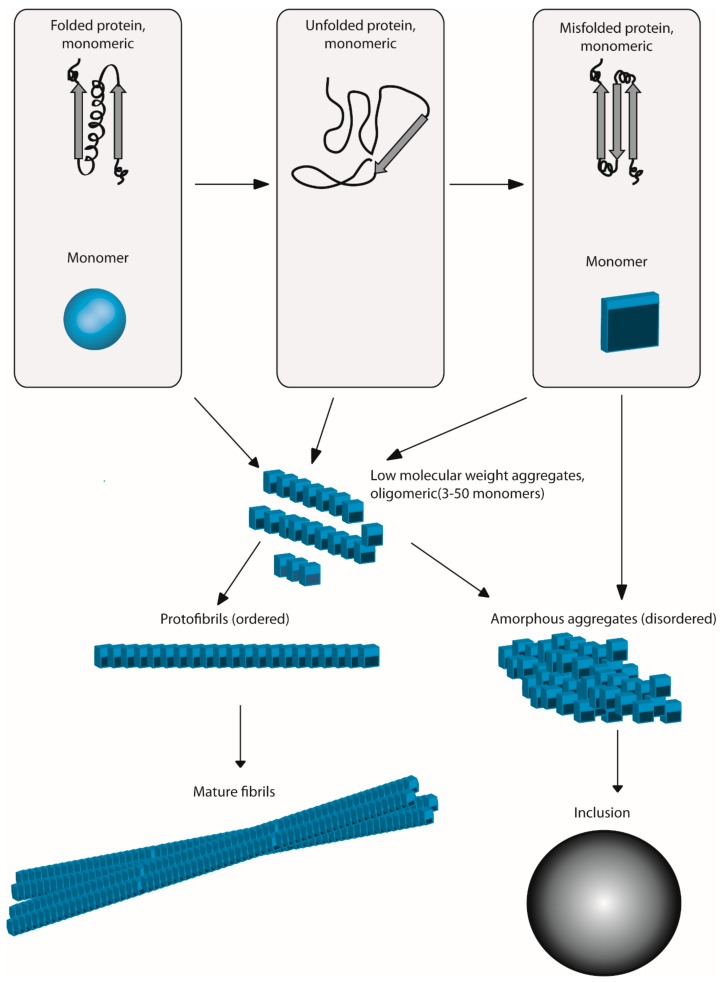
General picture of the protein aggregation process. The unfolded and misfolded monomer structures are aggregation prone. Folded monomers can also aggregate from native-like conformations without going through the unfolded step. The association of several monomers gives rise to oligomeric aggregates with low molecular weight. The addition of oligomers in an ordered manner permits the growth of oligomers to protofibrils and mature fibrils. Amorphous aggregates can arise from the precipitation of monomers or oligomers, possibly leading to protein inclusions.

Protein aggregation consists of any association of proteins into larger structures with non-native conformation [1]. Aggregates can have either an amorphous or a highly ordered structure (amyloid). The presence of aggregates is generally indicative of proteostasis imbalance, either due to insufficient proteostasis or to disrupted chaperone capacity. Aggregates can also be a response to cellular stress related to environmental changes [2] (Figure 1).

The intrinsic parameters of protein aggregation are not fully understood, but the ability of certain proteins to aggregate more readily compared to others has been known for a while. Intrinsic aggregation capacity can be obvious, as in the case of peptide poly(Q) tracts that form high molecular weight aggregates in some neurodegenerative diseases (including Huntington’s disease). Such aggregates exhibit a fibrillar or ribbon-like morphology, reminiscent of prion rods and amyloid-β (Aβ) fibrils in Alzheimer’s disease (AD) [3]. Aggregation is controlled in vitro, not only by poly(Q) tracts length (51–122 glutamines causing huntingtin’s aggregation in vitro), but also by protein concentration and reaction time [4]. In addition, most proteins contain one or more aggregation prone-regions (APR), which are protected from aggregation by protein interactions or by specific structural features (e.g., burying into a hydrophobic core) in physiological conditions [5].

However, the aggregation of proteins without any obvious features is intriguing. An interesting study used three different stress agents: Arsenite, a toxic metalloid, hydrogen peroxide (H_2_O_2_), a ubiquitous oxidative stress agent, and azetidine-2-carboxylic acid (AZC), a proline analog whose incorporation into proteins provokes conformation alterations, misfolding, and aggregation [6,7]. The authors identified that despite distinct stress conditions with distinct mechanisms of action, some of the proteins that aggregate are of similar types, suggesting that proteins within aggregates are intrinsically aggregation-prone [7]. Indeed, it appears that aggregation prone proteins like PrP^C^ (the prion protein) or Shadoo (also a member of the prion protein family) exhibit large intrinsically disordered domains (IDDs). The resulting conformational plasticity is likely to offer a wide range of interacting possibilities for these proteins, thereby regulating their localization and aggregation propensity [8,9,10]. Several computational approaches have tried to question the intrinsic determinisms of aggregation. Noticeably, the Waltz algorithm was developed to try to predict amylogenic regions in protein sequences, based on a scoring matrix deduced from the biophysical and structural analysis of previously characterized hexapeptides with amyloid properties [11]. Based on the accuracy of their pWALTZ prion prediction method derived from Waltz, Sabate and co-workers recently suggested a model for prion formation that depends on the presence of specific short sequence elements, embedded in intrinsically Q/N-rich regions, with high amyloid propensity [12].

Interestingly, the aggregation of globular proteins into amyloids from a native or native-like state has also been described. In such cases, aggregation-prone states can be reached from native-like conformations after small temperature or pH changes, or stress modulations in general, without the need to cross the energy barrier to unfold [13]. Several examples in the literature illustrate this fact, such as the aggregation of one of the acylphosphatases from the *Drosophila melanogaster* (AcPDro2) [13], the human lysozyme aggregating through a nucleation-dependent growth process [14], the globular acylphosphatase from *Sulfolobus solfataricus* (forming aggregates in which the monomers maintain their native-like topology [15]), and the transthyretin-like domain of human carboxypeptidase D (h-TTL), a monomeric protein with homology to human transthyretin that aggregates under close to physiological conditions [16]. Another example is the src tyrosine kinase SH3 domain, whose aggregation-prone state favors a domain swap that allows amyloid formation [17]. In such cases, the free energy gap between the native and the aggregation-prone state is an essential determinant of the aggregation propensity of the proteins [14,17]. Moreover, even though unfolded proteins have a high capacity to aggregate, the residual aggregation potential of proteins within a folded state may be physiologically relevant and play a role in several clinical situations [13,14,16].

Protein aggregation is obviously related to several human pathological situations. Amyloidoses, for example, are a group of diseases originating from the aggregation of oligomers into amyloid fibrils that deposit in tissues and are, in turn, toxic to the cells. Which of the oligomers or the fibrils are the most toxic to cells is still a matter of debate. Further, well-known neurodegenerative diseases, including Alzheimer’s (AD), Parkinson’s (PD), and motor neuron diseases, appear to be a consequence of protein aggregation in neurons, leading to toxicity and neuronal cell death. Although pathological protein aggregation may have different causes, the chronic disturbance of cellular homeostasis (due to exogenous pollutants, or aging for example) is likely to play a major role by modifying the physico-chemical equilibrium and influencing protein folding and aggregation. Redox perturbations, in particular, have long been linked to protein aggregation diseases.

Reactive oxygen species (ROS) are normal by-products arising from various cellular reactions, mostly during electron transport in mitochondria or chloroplasts. Intracellular ROS levels are maintained low within cells, ensuring redox homeostasis for proper cellular chemical reactions. Oxidative stress occurs when the ROS concentration is excessive regarding the antioxidant capacities of the cell, leading to the oxidation of cellular molecules and their alteration [18]. Proteins appear to be a major target for oxidation due to their elevated quantities compared to other cell components and also due to their high reactivity with ROS [19]. Proteins are susceptible to ROS modifications of amino acid side chains that alter their structure.

We provide here a review of recent data on the functional links between oxidative stress and protein aggregation.

## 2. Oxidative Stress Can Produce Aggregation: A Mechanistic View

### 2.1. Oxidation of Critical Amino Acids Induces Structural Changes within Proteins, Leading to Aggregation

#### 2.1.1. Cysteine Oxidation

Among the amino acids, cysteine (Cys) possesses a thiol group, which is highly nucleophilic. This structural feature makes cysteine particularly prone to oxidation by ROS. Amorphous aggregation of γ-cristallins is the cause of cataracts, a widespread disease among seniors. Internal disulfide bond formation between Cys32 and Cys41 due to oxidation was found necessary and sufficient to provoke aggregation under physiological conditions, likely by stabilizing an unfolded intermediate prone to protein–protein interaction between an extruded hairpin and a distal β-sheet in the γD-crystallin [20].

Being essential for serotonin synthesis, Tryptophan hydroxylase 2 (TPH2) is considered a phenotypic marker for serotonin neurons. Known to be extremely labile to oxidation, TPH2 aggregates through both intra- and inter-molecular disulfide cross-linking upon oxidation of cysteines [21]. Accordingly, in a systematic cysteine-mutagenesis approach, a single cysteine out of 13 was found sufficient for aggregation, whereas only cysteine-less mutants were found resistant to aggregation upon oxidation, thereby indicating that cysteines are necessary for the responses of TPH2 to oxidation. These results led the authors to hypothesize that redox homeostasis changes occurring during Parkinson’s disease might be involved in disulfide links in TPH2, causing TPH2 to shift from a soluble compartment to large inclusion bodies, consequently losing its catalytic function [21].

Alternatively, disulfide bonds can occur in proteins’ native structures in physiological conditions. Disulfide-rich domains (DRDs) are peptide domains whose native structures are stabilized by covalent disulfide bonds through an oxidative folding reaction. The authors questioned whether this specific folding might be associated with an increased aggregation propensity of DRDs. Among the 97 DRDs analyzed in silico, a majority were intrinsically disordered, but remained more soluble and had fewer aggregating regions than those of other globular domains [22]. This work suggests that DRDs might have evolved to avoid aggregation before proteins acquire their covalently linked native structures or after oxidative stress.

#### 2.1.2. Other Aminoacids Involved

Caseins are very abundant in milk as they represent more than 80% of total milk protein content [23]. Inter- and intra- covalent di-tyrosine (di-Tyr) and di-tryptophan (di-Trp) cross-links have been shown to provoke α- and β-casein aggregation, due to the Trp-or Tyr- derived radicals produced by photo-oxidation mediated by riboflavin, a photosensitizing vitamin [24]. Oxygen was found to strongly modulate this phenomenon and to increase protein aggregation by decreasing the overall cross-link formation, but allowing the formation of oxidized Trp, Tyr, Methionine (Met), and Histidine (His) residues [24].

Glyceraldehyde-3-phosphate dehydrogenase (GAPDH) is involved in energy production and has been shown to convert from its native soluble state into a non-native high molecular weight, which is insoluble, and an aggregated state, during the course of several diseases, including Alzheimer’s disease [25,26,27]. Interestingly, methionine, rather than cysteine oxidation, was shown to be a primary cause of GAPDH aggregation, as mutating methionine 46 to leucine rendered GAPDH highly resistant to aggregation after exposure to (3E)-4-ethyl-2-hydroxyimino-5-nitro-3-hexenamide (NOR3), a potent oxidative agent driving GAPDH aggregation [25,28,29]. In this case, the authors propose that methionine oxidation represents a “linchpin”, a permissive event for subsequent misfolding and aggregation [28].

It appears that protein residue oxidation, including the resulting disulfide bonds between cysteines, is not directly responsible for aggregation, per se, but in most cases induces limited or extensive unfolding of the surrounding environment of the protein. This further favors protein–protein interactions and aggregation, as summarized in Table 1.

### 2.2. Role of Metals

Metals’ contributions to Fenton’s or Haber-Weiss’ reactions have been described for a number of years, as they lead to highly reactive ROS that can be deleterious to cell components. In particular, they are responsible for amino acid oxidation. The involvement of metals in various pathological situations has also been recognized [30].

Parkinson’s disease pathology involves misfolding and aggregation of the presynaptic protein α-synuclein, together with an alteration of the homeostasis of brain metals, including iron. A recent analysis of iron’s role in the aggregation and secondary structure of N-terminally acetylated α-synuclein (^NAc^αS), the pathologically relevant form in PD, revealed the importance of oxidation in the phenomenon [31]. The sole addition of iron(II) in presence of oxygen was shown to induce an antiparallel right-twisted conformation of ^NAc^αS, and generate the oligomer-locked ^NAc^αS−Fe^II^/O_2_ conformation, thus initiating oligomer formation but preventing further processing into fibrils. This was not observed in the absence of oxygen. In contrast, the addition of iron(III) led to the formation of fibrils in the presence of oxygen. Thus, the iron oxidation status in the presence of oxygen differentially controls aggregation. This may have physiological or pathological implications [31].

Particular mutations in the Apolipoprotein A-I (APOA1) gene provoke hereditary amyloidosis. Interestingly, Fe(II) can reduce the formation of fibrillar APOA1 species, as opposed to Fe(III), which enhances their formation in vitro [32]. The increased levels in Fe(III) compared to Fe(II) under particular oxidative physiological conditions, e.g., in older patients or in presence of air pollutants, might thus contribute to the development of amyloidosis and possibly other diseases involving protein aggregation [32].

**Figure 2:**
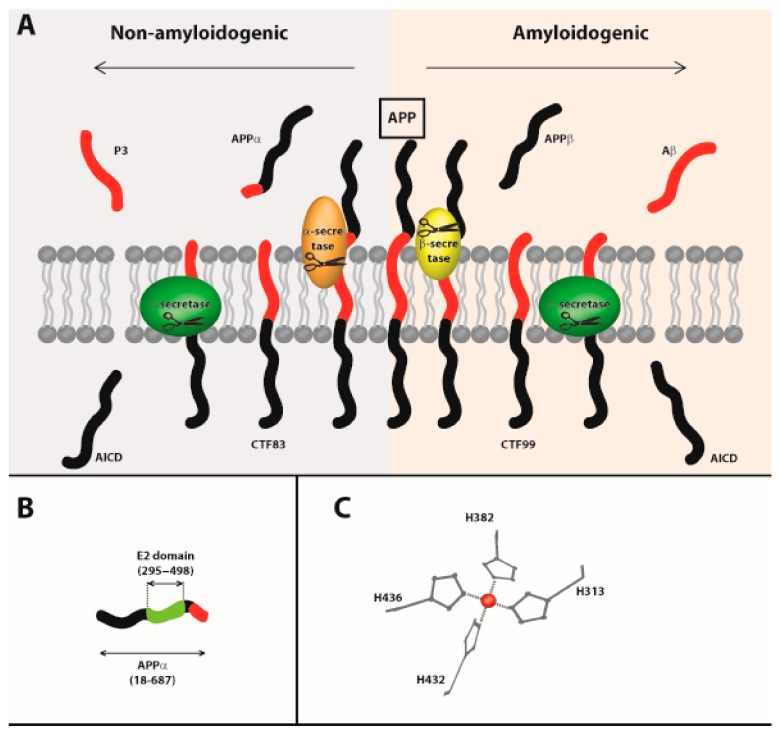
Amyloid Precursor Protein (APP) processing. (**A**) Cleavage of APP occurs through two pathways. The non-amyloidogenic pathway (grey, left) involves two cleavages by α- and γ-secretases and produces a long APPα fragment, which is secreted. C-Terminal Fragment (CTF)83, 3-kd peptide, (P3), and APP intracellular domain (AICD) fragments are also released. In parallel, the amyloidogenic pathway (pink, right) involves two cleavages by β- and γ-secretases, producing a long APPβ, which is secreted. CTF99, AICD, and Aβ fragments are also produced. In pathological conditions, Aβ peptides accumulate and can ultimately aggregate and form oligomers and fibrils that are toxic for the cells (adapted from [36]). (**B**) Schematic representation of the APPα fragment, produced after cleavage of the APP by α-secretase. The E2 domain is highlighted (green). (**C**) Zooming in on the four cysteine residues involved in the tetra-His M1 site, included in the E2 domain. The Cu cation is pictured as a red ball (adapted from [37] with permission).

Several studies have demonstrated amyloid-β-mediated ROS production in the presence of Cu ions, and mechanisms that can generate a superoxide anion (O_2_• −), hydrogen peroxide (H_2_O_2_), and a hydroxyl radical (HO•) have been proposed [33,34]. The Amyloid-β peptide (Aβ) is produced after cleavage of the transmembrane Amyloid Precursor Protein (APP) by β- and γ-secretases. Aβ is thus released in the extracellular space. Its accumulation and aggregation are a hallmark of Alzheimer’s disease (See Figure 2). A recent work identified that Cu(I) or Cu(II) cations predominantly bind the M1 site located in domain E2 of APP, with a comparablely high affinity (picomolar) being liganded by four histidine residues. Cu(II) binding to M1 was found to stabilize E2 but also alters its structural conformation into a more open state [35]. The authors demonstrated the aerobic catalytic oxidation of ascorbate, an abundant antioxidant molecule in the central nervous system, by the Cu-E2 complex, with an experimental set up close to physiological conditions. APPβ might thus play the role of redox catalyst in vivo and cause protein damage, as observed in brain tissues from Alzheimer’s patients [35].

### 2.3. Carbonylation Leads to Aggregation

Carbonylation is a particular type of oxidation involving the irreversible addition of a carbonyl group (CO) into proteins. The ROS-mediated carbonylation of proteins mainly affects lysine, arginine, threonine, and proline residues [38] and was frequently reported in chronic inflammatory diseases [39,40]. Protein carbonylation is commonly considered a standard marker of oxidative stress. While carbonylation is reported as having a role in protein quality control, in tagging damaged proteins for degradation with the 20S proteasome (via an unresolved mechanism), excessive carbonylation has been shown to be responsible for protein aggregation, especially when proteasome activity is impaired [41]. However, few publications provide mechanistic insight into how protein carbonylation causes aggregation. A recent report indicated that these specific modifications are also associated with physiological aging and favor the formation of denser and more compact aggregates, including several proteins previously reported for their aggregation propensity [42]. Inducing carbonyl stress in young mice increased protein aggregation, similar to the aggregation naturally observed in old mice, indicating that post-translational oxidative alterations are responsible for increased protein aggregation [42]. Despite evidence that carbonylation leads to aggregation, to our knowledge, no direct mechanism has been explicitly reported.

Recent attention has been paid to reactive dicarbonyl species, such as methylglyoxal (MGO) and glyoxal (GO), considered to be side-products of several metabolic pathways, as they produce specific oxidative modifications of proteins, mainly reacting with lysine and arginine [43]. These oxidative modifications, including carbonylation, are subsequently responsible for secondary and tertiary structure alterations and the formation of high molecular mass protein aggregates and may have an underestimated role in several proteinopathies, including Alzheimer’s and Parkinson’s diseases.

### 2.4. Protein Oxidation Influences Aggregation by Modulating Chaperone Protein Activity

Oxidation-induced structural changes in proteins may also prevent specific interactions with partners. A very interesting case has been described recently, with the nucleotide exchanger and co-chaperone Mge1 within the mitochondria. Mge1 is actually modified by persistent oxidative stress, and methionine 155, in particular, can be reversibly oxidized into methionine sulfoxide. As a result, due to local structural changes in Mge1 binding to its partner, the heat shock protein 70 (Hsp70) is defective. Hsp70 is a central heat-shock protein involved in controlling protein folding and plays an important role in ensuring proteostasis. If not reduced by the endogenous methionine sulfoxide reductase, the oxidized Mge1 aggregates into amyloid-type particles. Interestingly, the authors noticed that highly oxidized Mge1 actually increased the binding capacity of Hsp70 to a denatured protein, suggesting that the oxidation-induced defective binding of Meg1 to Hsp70 and MgeI aggregation may protect the cells from the aggregation of other proteins in an oxidative stress context [44]. It is interesting to note that some isoforms of Hsp70 are stress-modulated. For instance, oxidants like methylene blue and hydrogen peroxide inactivate Hsp72, possibly due to the oxidation of two specific cysteine residues resulting in structural changes within the nucleotide-binding domain. Noticeably, this oxidation-induced inactivation of Hsp72 is associated with decreased levels of tau in several Alzheimer’s disease models. The authors suggest that Hsp72 inactivation could clear the cytosol from misfolded Hsp72 substrates like tau, even though the mechanism remains unclear [45].

Tsa1 is a surprising protein that exhibits a double role, acting as a peroxidase but also as a chaperone for aggregated proteins—specifically, by chaperoning misassembled ribosomal proteins, thereby preventing toxicity to arise from aggregation [46]. It is noticeable that the absence of Tsa1 both provokes an increase in [H_2_O_2_] and the accumulation of aggregated proteins in the meantime [47]. This dual role might argue for a strong functional connection between oxidative stress and protein aggregation.

### 2.5. Protein Oxidation Influences Aggregation by Perturbing the Translational Process

Yeast Sup35 is a translation termination factor well known for its capacity to form a prion (i.e., a self-perpetuating amyloid aggregate), in response to environmental or cellular factors. This prion is called [*PSI+*] [48]. When aggregated into [*PSI+*], Sup35 loses its function in translation termination, and an elevated read-through of the stop codons occurs, thereby generating C-terminally extended polypeptides. Following exposure to H_2_O_2_, [*PSI*^+^] formation was shown to occur with increased frequency, possibly linked to the oxidation of methionine residues [49]. As a consequence, exposure to oxidative stress perturbs the translational process through titration of Sup35.

Defective mRNAs are normally handled by mRNA quality control systems within cells, among which the non-stop decay (NSD) pathway and the no-go decay (NGD) pathways prevent the production of abnormal, potentially aggregation-prone proteins. Interestingly, NSD components (e.g., Ski7) and NGD components (e.g., Dom34/Hbs1) were recently shown to be required during oxidative stress [50]. Moreover, overexpression of Sup35 actually decreases stop codon read-through and improves the tolerance of yeast cells to oxidative stress, thus providing an unanticipated link between oxidative stress tolerance and NSD, as NSD substrates noticeably accumulate as a consequence of [*PSI*^+^] formation after oxidative stress [50].

### 2.6. Oxidative Stress Contributes to Aggregation by Modulating the Proteasome and Autophagy Capacity

Proteasomes are central players of the Ubiquitin–Proteasome System (UPS), which ensures the quality control of proteins. The 20S proteasome, constituting 28 subunits, is considered the “core” proteasome and is involved in unfolded, misfolded, or intrinsically disordered or oxidized protein removal by proteolytic degradation. Ubiquitination is not needed for degradation by the 20S proteasome, unlike the 26S proteasome. Several other proteasome components regarded as regulators have been characterized, including the 19S proteasome. Obviously, increasing 20S proteasome activity in pathogenic conditions due to protein aggregation might be of interest. The small, imidazoline-derivative molecule TCH-165 was shown to increase 20S proteasome levels to the detriment of the 26S proteasome, resulting in the enhanced proteolysis of intrinsically disordered proteins. These include aggregation-prone proteins such as α-synuclein and tau, whose aggregation are hallmarks of Parkinson’s and Alzheimer’s diseases, respectively, and proto-oncogenes, such as ornithine decarboxylase and c-Fos. Noticeably, high concentrations of this molecule resulted in the accumulation of ubiquitinated substrates [51].

Similarly, proteasome impairment has been shown to favor the accumulation and aggregation of aggregation-prone proteins [52,53,54,55]. UPS disturbances have been correlated with the spreading of diseases involving protein aggregation, including Alzheimer’s, Parkinson’s, and Huntington’s diseases, as well as amyotrophic lateral sclerosis (ALS). ALS, in particular, is characterized by ubiquitinated proteic inclusions.

For our current focus, it is worth noticing that the UPS is actually redox regulated, and S- glutathionylation, specifically, has been involved in the post-translational control of proteasome activity. For example, 20S glutathionylation was shown to be responsible for increased proteolytic activity [56], favoring the removal of oxidized or unstructured proteins in stressful situations. After an acute oxidative event, a coordinated response involving poly[ADP-ribose] polymerase 1 (PARP-1) activation and a ubiquitination block, followed by the inactivation of the proteolytic capacity, occurs, before massive de novo synthesis of proteasome players and consequently increased proteolytic activity [57]. In contrast to 20S, 26S proteolytic activity was reduced in oxidative conditions. Indeed, the S-gluthationylation of a regulatory subunit of the 19S proteasome, resulting in diminished 26S proteasome activity, was observed in the presence of chronically increased hydrogen peroxide concentrations both in vitro and in cellulo [58]. These data might reveal differential redox regulations for the proteasome’s components. Under normal physiological conditions, the oxidized proteasomes (ubiquitin-dependent proteolytic 20S core and regulatory 19S) are S-glutathionylated, as evidenced in several cellular contexts [56,58,59,60].

It is also interesting to report here that the anti-malaria drug dihydroartemisinin, an artemisinin derivative used in clinics, acts by inhibiting the parasite’s proteasome, possibly through oxidative damage to the proteasome [61].

**Figure 3:**
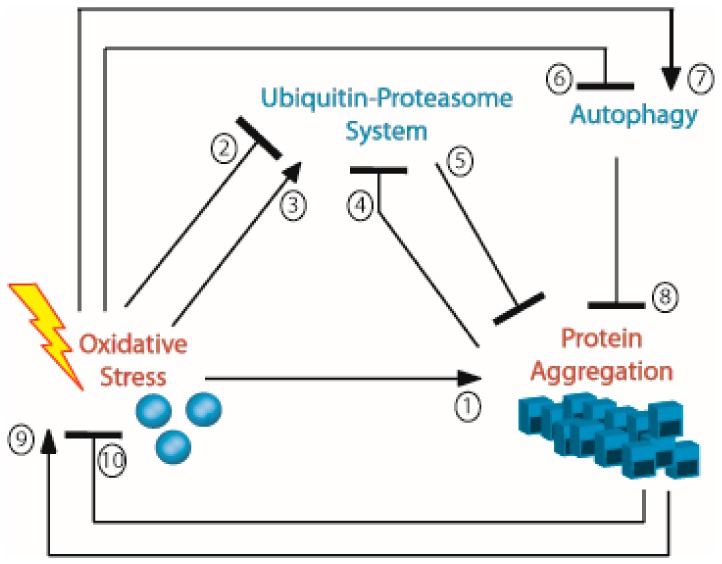
General diagram showing the regulation of protein aggregation. Arrows indicate the activation relationship, and blunt-ended arrows indicate the repression relationship. References supporting this diagram within this review are as follows: 1: [20,21,24,28,31,32,35,41,42,43,44,45,46,47,49,50]; 2: [58,61]; 3: [57]; 4: [62,63]; 5: [51,52,53,54,55]; 6: [80]; 7: [74,75,76,77,78,79,80]; 8: [64,65,66,67,68,69,70,71,72,73,82,83,84]; 9: [47,88,89,90,91,92,93,94,95,96,97,98,99]; 10: [100,101,102,103,104,105,106,107].

Thus, chronic oxidative stress favors protein aggregation through by impairing proteasome capacity (Figure 3). In addition, the slow and gradual accumulation of aggregates during aging has been observed. These aggregates are composed of oxidized proteins (including carbonylated aggregates) due to proteins escaping the UPS and are suggested to bind the proteasome and inhibit its function [62,63], thereby fostering this aggregation loop.

Autophagy is a cellular process that allows the recycling of cellular components, such as proteins or organelles, through a lysosome-mediated catabolic pathway. Particular types of autophagy are primarily distinguished (macroautophagy, chaperone-mediated autophagy, microautophagy) by how target substrates are recognized and delivered to lysosomes. More specifically, autophagy is central in eliminating the substrates that will ultimately give rise to amyloids and fibrils in several neurodegenerative diseases (Aβ peptide and tau protein in AD [64,65], alpha-synuclein in PD [66], mutated huntingtin in Huntington disease [67], and superoxide dismutase 1 (SOD1) in ALS [68]). Accordingly, mice deficient in autophagy show neurodegenerative disorders, and defects in autophagy have been associated with various human neurodegenerative diseases [69,70,71,72,73].

Redox regulation of autophagy has been suggested, as antioxidant treatments actually prevent autophagy [74]. This type of regulation was proposed by several authors [75,76,77,78,79]. Interestingly, recent work identified that the absence of the glutathione reductase *gsr-1* gene leads to major redox homeostasis unbalance in the model organism, *Caenorhabditis elegans*, and is also responsible for autophagy impairment by preventing the nuclear translocation of a key transcription factor, HLH-30/TFEB. In addition, the aggregation of both homologous and heterologous proteins (Aβ peptide (AD), α-synuclein:: YFP (PD), and Q40::YFP (Huntington disease) expressed in *C. elegans* was increased. This study reveals a glutathione-dependent regulation of autophagy, allowing the control of protein aggregation, a process also conserved from lower eukaryotes to mammals [80]. Recently, several drugs were developed to modulate autophagy in search of therapeutic improvement in neurodegenerative disorders. Noticeably, Rapamycin targets mTor, a master regulator of cell growth and metabolism. Rapamycin activates autophagy and lysosomal biogenesis [81] and, furthermore, was shown capable of reducing Aβ accumulation and improving cognitive impairments in a transgenic mouse model by increasing autophagy [82,83]. Autophagy activation by rapamycin in neurons was also shown to favor the clearing of intracellular aggregates of misfolded prion proteins and to reduce neurotoxicity [84]. The rapamycin derivative, Rilmenidine, which is protective against oxidative cytotoxicity [85], was also shown to increase autophagy, despite failing to decrease the accumulation and aggregation of SOD1 in a mouse ALS model [86]. Another rapamycin derivative, biolimus, was also shown to be capable of activating autophagy efficiently in smooth muscle cells [87]. Most of these drugs have been shown to play on the oxidative balance within cells. Nevertheless, whether the effect of these drugs on protein aggregation is related to redox modulation has not been consistently characterized.

## 3. Aggregation Can, in Turn, Produce Oxidative Stress, or Protect Against Oxidative Insults

### 3.1. Pro-Oxidative Effects

As noticed previously, the yeast peroxiredoxin Tsa1 acts both as a chaperone and an ROS scavenger. The proline analogue azetidine-2-carboxylic acid (AZC) induces aggregation in yeast cells, and yeast mutants lacking *TSA1* are highly sensitive to AZC-induced misfolding. The toxicity of AZC is actually related to ROS accumulation, as decreasing ROS levels prevents sensitivity to AZC. Interestingly, inhibiting nascent protein synthesis with cycloheximide rescues the *tsa1* mutant sensitivity to AZC, confirming that aggregation in this case causes ROS production [47].

ROS production due to mitochondrial dysfunction was often found to be associated with protein aggregation, as in the case of Huntington’s [88,89] or Alzheimer’s disease [90]. Alpha–synuclein aggregation is a hallmark of Parkinson’s disease, as previously explained, and is known to influence mitochondrial morphology, interrupting ER–mitochondria communication [108,109,110] and modulating mitochondrial fragmentation [111], even though the molecular determinants of these changes are not very clear. The preferential binding of pathological alpha–synuclein aggregates to mitochondria was recently identified in neurons [91], and this process was accompanied by cellular respiration defects, suggesting mitochondrial dysfunction and leading to the hypothesis of a direct mitochondrial impairment by alpha-synuclein aggregates, inducing ROS production.

RNA aptamers are synthetic nontoxic and non-immunogenic RNA oligonucleotides able to bind a specific target and can thus be used as therapeutic weapons. For instance, specific RNA aptamers can efficiently inhibit aggregation of mutant huntingtin, with a pathogenic polyglutamine stretch, by stabilizing the monomer both in vitro and in cellulo. Oxidative stress is a hallmark of Huntington’s disease and has been shown to occur in cells exposed to mutant huntingtin aggregates. Whether it is a cause or a consequence of the disease is not yet clear. However, it is interesting to note that RNA aptamers inhibiting mutant huntingtin’s aggregation lead to reduced oxidative stress levels in cellular models [92]. The authors smartly engineered cells expressing RNA aptamers under the control of an oxidative stress-inducible promoter. A nine-fold increase in aptamer expression occurred in mutant huntingtin-expressing cells, which consequently reduced mutant huntingtin aggregation in these cells. As a consequence, oxidative stress levels were also reduced, so RNA aptamer expression was also, in turn, reduced [93]. This smart design unambiguously demonstrates that protein aggregation is responsible for intracellular oxidative stress production in this context. Accordingly, mitochondrial dysfunction was also reduced using this particular experimental design, indicating that ROS production is likely related to mitochondrial impairment [93].

Human Amylin (hA) is a 4 kDa pancreatic hormone, synthesized and secreted along with insulin by islet beta cells. Like other amyloid proteins, it is prone to aggregation and remains a hallmark of type-2 diabetes mellitus [96]. Previous studies indicated that either exposure of beta cells to hA or hA-overexpression in cells results in intracellular ROS accumulation, supporting the hypothesis that hA aggregation might be a cause of oxidative stress [94], possibly through mitochondrial dysfunction [95]. Recent work identified that a misfolded amylin actually activates an upstream apoptosis signal regulating kinase-1 (ASK1), with a concomitant decrease in the intracellular levels of reduced glutathione [96]. Moreover, the pro-oxidative activity and expression of a plasma membrane bound NADPH oxidase (NOX) and its regulatory subunits were stimulated, suggesting that NOX1 and ASK1 mediate the cytotoxic effect of aggregated hA in pancreatic beta-cells [96]. Interestingly, NOX had also been previously identified as a main ROS producer in cultured neurons exposed to amyloid-β peptides [97,98].

Stabilizing α-synuclein monomers also proved to decrease the growth of misfolded cytotoxic aggregates and consequently reduced oxidative damage to the cells by limiting the binding of aggregates to the cell membrane [99]. Thus, a membrane’s structure seems to play an important role in oxidative stress production in response to protein aggregation, possibly involving membrane bound oxidases for ROS production, as a consequence of aggregates binding to the membrane.

### 3.2. Anti-Oxidative Effects

Even though the role of aggregates in ROS production was unambiguously demonstrated through obvious examples, other findings describe how aggregates can also “buffer” oxidative effects. Surprisingly, 40-aminoacids, as well as 28-aminoacids amyloid-β peptides (Aβ_1–40_ or Aβ_1–28_), either soluble or aggregated, revealed potent anti-oxidant activity in cell-free systems, despite their accumulation and aggregation being a hallmark of Alzheimer’s disease, which is also characterized by chronic oxidative stress [100,101]. The authors suggest that peptides might first chelate metal ions, including Zn(II), Cu(II), and Fe(II), as previously suggested by other authors [102,103,104], which would inhibit Fenton’s reaction, and then scavenge radicals by oxidation of His and Tyr residues [100,101]. Whether a peptide with similar His and Tyr residue content are also capable of “buffering” free radicals would be worth testing. A more recent study reported similar anti-oxidant activity of amyloid-β aggregates. Overexpression of 21 variants of the amyloid-β 42 peptide fused with GFP in yeast (covering a broad range of intrinsic aggregation propensities) led to various levels of intrinsic oxidative stress production [105]. A striking correlation was observed—the more aggregation-prone the mutated GFP-Abeta is, the less oxidative stress is produced, suggesting that large insoluble aggregates might act to limit cellular oxidative stress. An aggregation propensity threshold could be defined, above which proteins with a high aggregation propensity accumulate into foci. The authors then suggested that protein foci formation is an active ATP-dependent process, which might serve as a protective mechanism against oxidative stress damage, despite its high energetic price [106].

Similarly, it is noteable that [*PSI+*] formation has been reported as protective against oxidative stress, as antioxidant enzyme-lacking yeasts become more sensitive to H_2_O_2_ when [*PSI+*] is eliminated [107].

Finally, Carija et al. propose that different kinds of aggregates have different effects on ROS levels, as small particles called “diffuse” aggregates are associated with increased intracellular oxidative stress, whereas larger protein inclusions are not [105]. Similar conclusions were previously reached, that hydrogen peroxide is generated during the early stages of protein aggregation in the course of Alzheimer’s disease pathogenesis, possibly by an early form of protein aggregation, in the absence of a mature amyloid fibril [112].

## 4. Aggregation and Oxidation can be Parts of a Vicious Circle

In about 12% of familiar and 1.5% of sporadic cases of ALS, mutations in the superoxide dismutase 1 (SOD1), a major player in ROS detoxification, are found. The misfolding and aggregating of SOD1 are considered a hallmark of the disease [113]. A mutated version of SOD1 named SOD1^A4V^ is particularly aggregation-prone. Its expression is associated with a profound disturbance of free ubiquitin distribution within the cells, and with UPS dysfunction. These data suggest that protein aggregation, per se, might cause UPS disturbance and participate in a vicious circle that eventually prevents the elimination of aggregates [114]. Because SOD1 is a major player in ROS detoxification, and its absence is responsible for increased oxidative stress, the latter being suggested to play a key role in ALS progression [115,116], it is a possibility that oxidative stress plays a role in UPS disturbance in this particular case. Both would participate a vicious circle involving SOD1 aggregation and oxidative stress.

In light of the mechanistic insights described previously, multifunctional modulators that are able to chelate metals and prevent ROS generation and protein aggregation have been designed for several years, in an attempt to develop new therapeutic tools [117,118]. TGR86 is composed of both the metal chelating clioquinol and the antioxidant, epigallocatechin gallate [119]. TGR86 was shown to be capable of both interacting with amyloid-β and efficiently modulating its aggregation and complexing with Cu(II), as well as preventing ROS generation. Another bifunctional molecule, composed of a nitroxide spin label linked to an amyloidophilic fluorine (spin-labeled fluorine, SLF), was recently developed [120]. Using super-resolution confocal microscopy imaging, the authors identified that assembling those two modules within SLF creates a synergistic effect in cultured neurons, preventing the intracellular accumulation of amyloid-β on the one hand, together with the reduction and the scavenging of amyloid-β-induced ROS on the other. Further studies are now needed to evaluate the therapeutic potential of these molecules in Alzheimer’s disease.

## Figures and Tables

**Table 1:** Residue oxidation causing protein aggregation.

Protein(s) Involved	Mechanism	Residue Involved	Related Disease/Pathway	Reference
γD-crystallin	Cys32–Cys41 internal disulfide bond formation leads to the stabilization of a partially unfolded domain, which is prone to further intermolecular interactions. Internal disulfide bond formation provokes aggregation under physiological conditions.	Cysteine	Cataracts in older people	[20]
Tryptophan hydroxylase 2 (TPH2)	Intra- and inter-molecular disulfide bonds responsible for high molecular weight aggregates.	Cysteine	Parkinson’s disease	[21]
Milk caseins	Oxidation of Tryptophan, Tyrosine, Methionine, and Histidine residues decreases di-Tyr and di-Trp formation but allows increased protein aggregation.	Tryptophan, Tyrosine, Methionine, Histidine	None	[24]
Glyceraldehyde-3-phosphate dehydrogenase (GAPDH)	Oxidation of Methionine allows subsequent misfolding and further aggregation of GAPDH.	Methionine	None	[28]

## References

[1] Hipp M.S., Park S.H., Hartl F.U. (2014). Proteostasis impairment in protein-misfolding and -aggregation diseases. Trends Cell Biol..

[2] Li J., Labbadia J., Morimoto R.I. (2017). Rethinking HSF1 in Stress, Development, and Organismal Health. Trends Cell Biol..

[3] Scherzinger E., Lurz R., Turmaine M., Mangiarini L., Hollenbach B., Hasenbank R., Bates G.P., Davies S.W., Lehrach H., Wanker E.E. (1997). Huntingtin-encoded polyglutamine expansions form amyloid-like protein aggregates *in vitro* and *in vivo*. Cell.

[4] Scherzinger E., Sittler A., Schweiger K., Heiser V., Lurz R., Hasenbank R., Bates G.P., Lehrach H., Wanker E.E. (1999). Self-assembly of polyglutamine-containing huntingtin fragments into amyloid-like fibrils: Implications for Huntington’s disease pathology. Proc. Natl. Acad. Sci. USA.

[5] Beerten J., Schymkowitz J., Rousseau F. (2012). Aggregation prone regions and gatekeeping residues in protein sequences. Curr. Top. Med. Chem..

[6] Trotter E.W., Berenfeld L., Krause S.A., Petsko G.A., Gray J.V. (2001). Protein misfolding and temperature up-shift cause G1 arrest via a common mechanism dependent on heat shock factor in Saccharomycescerevisiae. Proc. Natl. Acad. Sci. USA.

[7] Weids A.J., Ibstedt S., Tamas M.J., Grant C.M. (2016). Distinct stress conditions result in aggregation of proteins with similar properties. Sci. Rep..

[8] Ciric D., Richard C.A., Moudjou M., Chapuis J., Sibille P., Daude N., Westaway D., Adrover M., Beringue V., Martin D. (2015). Interaction between Shadoo and PrP Affects the PrP-Folding Pathway. J. Virol..

[9] Pepe A., Avolio R., Matassa D.S., Esposito F., Nitsch L., Zurzolo C., Paladino S., Sarnataro D. (2017). Regulation of sub-compartmental targeting and folding properties of the Prion-like protein Shadoo. Sci. Rep..

[10] Sarnataro D. (2018). Attempt to Untangle the Prion-Like Misfolding Mechanism for Neurodegenerative Diseases. Int. J. Mol. Sci..

[11] Maurer-Stroh S., Debulpaep M., Kuemmerer N., Lopez de la Paz M., Martins I.C., Reumers J., Morris K.L., Copland A., Serpell L., Serrano L. (2010). Exploring the sequence determinants of amyloid structure using position-specific scoring matrices. Nat. Methods.

[12] Sabate R., Rousseau F., Schymkowitz J., Ventura S. (2015). What makes a protein sequence a prion?. PLoS Comput. Biol..

[13] Soldi G., Bemporad F., Torrassa S., Relini A., Ramazzotti M., Taddei N., Chiti F. (2005). Amyloid formation of a protein in the absence of initial unfolding and destabilization of the native state. Biophys. J..

[14] Dumoulin M., Kumita J.R., Dobson C.M. (2006). Normal and aberrant biological self-assembly: Insights from studies of human lysozyme and its amyloidogenic variants. Acc. Chem. Res..

[15] Bemporad F., Vannocci T., Varela L., Azuaga A.I., Chiti F. (2008). A model for the aggregation of the acylphosphatase from Sulfolobus solfataricus in its native-like state. Biochim. Biophys. Acta.

[16] Garcia-Pardo J., Grana-Montes R., Fernandez-Mendez M., Ruyra A., Roher N., Aviles F.X., Lorenzo J., Ventura S. (2014). Amyloid formation by human carboxypeptidase D transthyretin-like domain under physiological conditions. J. Biol. Chem..

[17] Zhuravlev P.I., Reddy G., Straub J.E., Thirumalai D. (2014). Propensity to form amyloid fibrils is encoded as excitations in the free energy landscape of monomeric proteins. J. Mol. Biol..

[18] Kim G.H., Kim J.E., Rhie S.J., Yoon S. (2015). The Role of Oxidative Stress in Neurodegenerative Diseases. Exp. Neurobiol..

[19] Ahmad S., Khan H., Shahab U., Rehman S., Rafi Z., Khan M.Y., Ansari A., Siddiqui Z., Ashraf J.M., Abdullah S.M. (2017). Protein oxidation: An overview of metabolism of sulphur containing amino acid, cysteine. Front. Biosci..

[20] Serebryany E., Woodard J.C., Adkar B.V., Shabab M., King J.A., Shakhnovich E.I. (2016). An Internal Disulfide Locks a Misfolded Aggregation-prone Intermediate in Cataract-linked Mutants of Human gammaD-Crystallin. J. Biol. Chem..

[21] Kuhn D.M., Sykes C.E., Geddes T.J., Jaunarajs K.L., Bishop C. (2011). Tryptophan hydroxylase 2 aggregates through disulfide cross-linking upon oxidation: Possible link to serotonin deficits and non-motor symptoms in Parkinson’s disease. J. Neurochem..

[22] Fraga H., Grana-Montes R., Illa R., Covaleda G., Ventura S. (2014). Association between foldability and aggregation propensity in small disulfide-rich proteins. Antioxid. Redox Signal..

[23] Jenness R. (1979). Comparative aspects of milk proteins. J. Dairy Res..

[24] Fuentes-Lemus E., Silva E., Leinisch F., Dorta E., Lorentzen L.G., Davies M.J., Lopez-Alarcon C. (2018). Alpha- and beta-casein aggregation induced by riboflavin-sensitized photo-oxidation occurs via di-tyrosine cross-links and is oxygen concentration dependent. Food Chem..

[25] Nakajima H., Amano W., Kubo T., Fukuhara A., Ihara H., Azuma Y.T., Tajima H., Inui T., Sawa A., Takeuchi T. (2009). Glyceraldehyde-3-phosphate dehydrogenase aggregate formation participates in oxidative stress-induced cell death. J. Biol. Chem..

[26] Cumming R.C., Schubert D. (2005). Amyloid-beta induces disulfide bonding and aggregation of GAPDH in Alzheimer’s disease. FASEB J..

[27] Tsuchiya K., Tajima H., Kuwae T., Takeshima T., Nakano T., Tanaka M., Sunaga K., Fukuhara Y., Nakashima K., Ohama E. (2005). Pro-apoptotic protein glyceraldehyde-3-phosphate dehydrogenase promotes the formation of Lewy body-like inclusions. Eur. J. Neurosci..

[28] Samson A.L., Knaupp A.S., Kass I., Kleifeld O., Marijanovic E.M., Hughes V.A., Lupton C.J., Buckle A.M., Bottomley S.P., Medcalf R.L. (2014). Oxidation of an exposed methionine instigates the aggregation of glyceraldehyde-3-phosphate dehydrogenase. J. Biol. Chem..

[29] Nakajima H., Amano W., Fujita A., Fukuhara A., Azuma Y.T., Hata F., Inui T., Takeuchi T. (2007). The active site cysteine of the proapoptotic protein glyceraldehyde-3-phosphate dehydrogenase is essential in oxidative stress-induced aggregation and cell death. J. Biol. Chem..

[30] Sayre L.M., Perry G., Atwood C.S., Smith M.A. (2000). The role of metals in neurodegenerative diseases. Cell. Mol. Biol..

[31] Abeyawardhane D.L., Fernandez R.D., Murgas C.J., Heitger D.R., Forney A.K., Crozier M.K., Lucas H.R. (2018). Iron Redox Chemistry Promotes Antiparallel Oligomerization of alpha-Synuclein. J. Am. Chem. Soc..

[32] Del Giudice R., Pesce A., Cozzolino F., Monti M., Relini A., Piccoli R., Arciello A., Monti D.M. (2018). Effects of iron on the aggregation propensity of the N-terminal fibrillogenic polypeptide of human apolipoprotein AI. BioMetals.

[33] Cheignon C., Jones M., Atrian-Blasco E., Kieffer I., Faller P., Collin F., Hureau C. (2017). Identification of key structural features of the elusive Cu-Abeta complex that generates ROS in Alzheimer’s disease. Chem. Sci..

[34] Cheignon C., Tomas M., Bonnefont-Rousselot D., Faller P., Hureau C., Collin F. (2018). Oxidative stress and the amyloid beta peptide in Alzheimer’s disease. Redox Biol..

[35] Young T.R., Pukala T.L., Cappai R., Wedd A.G., Xiao Z. (2018). The Human Amyloid Precursor Protein Binds Copper Ions Dominated by a Picomolar-Affinity Site in the Helix-Rich E2 Domain. Biochemistry.

[36] Thinakaran G., Koo E.H. (2008). Amyloid precursor protein trafficking, processing, and function. J. Biol. Chem..

[37] Dienemann C., Coburger I., Mehmedbasic A., Andersen O.M., Than M.E. (2015). Mutants of metal binding site M1 in APP E2 show metal specific differences in binding of heparin but not of sorLA. Biochemistry.

[38] Weng S.L., Huang K.Y., Kaunang F.J., Huang C.H., Kao H.J., Chang T.H., Wang H.Y., Lu J.J., Lee T.Y. (2017). Investigation and identification of protein carbonylation sites based on position-specific amino acid composition and physicochemical features. MC Bioinform..

[39] Arena S., Salzano A.M., Renzone G., D’Ambrosio C., Scaloni A. (2014). Non-enzymatic glycation and glycoxidation protein products in foods and diseases: An interconnected, complex scenario fully open to innovative proteomic studies. Mass Spectrom. Rev..

[40] Haigis M.C., Yankner B.A. (2010). The aging stress response. Mol. Cell.

[41] Castro J.P., Ott C., Jung T., Grune T., Almeida H. (2012). Carbonylation of the cytoskeletal protein actin leads to aggregate formation. Free Radic. Biol. Med..

[42] Tanase M., Urbanska A.M., Zolla V., Clement C.C., Huang L., Morozova K., Follo C., Goldberg M., Roda B., Reschiglian P. (2016). Role of Carbonyl Modifications on Aging-Associated Protein Aggregation. Sci. Rep..

[43] Bhat S.A., Bhat W.F., Afsar M., Khan M.S., Al-Bagmi M.S., Bano B. (2018). Modification of chickpea cystatin by reactive dicarbonyl species: Glycation, oxidation and aggregation. Arch. Biochem. Biophys..

[44] Karri S., Singh S., Paripati A.K., Marada A., Krishnamoorthy T., Guruprasad L., Balasubramanian D., Sepuri N.B.V. (2018). Adaptation of Mge1 to oxidative stress by local unfolding and altered Interaction with mitochondrial Hsp70 and Mxr2. Mitochondrion.

[45] Miyata Y., Rauch J.N., Jinwal U.K., Thompson A.D., Srinivasan S., Dickey C.A., Gestwicki J.E. (2012). Cysteine reactivity distinguishes redox sensing by the heat-inducible and constitutive forms of heat shock protein 70. Chem. Biol..

[46] Rand J.D., Grant C.M. (2006). The thioredoxin system protects ribosomes against stress-induced aggregation. Mol. Biol. Cell.

[47] Weids A.J., Grant C.M. (2014). The yeast peroxiredoxin Tsa1 protects against protein-aggregate-induced oxidative stress. J. Cell Sci..

[48] Wickner R.B. (1994). [URE3] as an altered URE2 protein: Evidence for a prion analog in Saccharomyces cerevisiae. Science.

[49] Doronina V.A., Staniforth G.L., Speldewinde S.H., Tuite M.F., Grant C.M. (2015). Oxidative stress conditions increase the frequency of de novo formation of the yeast [PSI+] prion. Mol. Microbiol..

[50] Jamar N.H., Kritsiligkou P., Grant C.M. (2017). The non-stop decay mRNA surveillance pathway is required for oxidative stress tolerance. Nucleic Acids Res..

[51] Njomen E., Osmulski P.A., Jones C.L., Gaczynska M., Tepe J.J. (2018). Small Molecule Modulation of Proteasome Assembly. Biochemistry.

[52] Matsui H., Ito H., Taniguchi Y., Inoue H., Takeda S., Takahashi R. (2010). Proteasome inhibition in medaka brain induces the features of Parkinson’s disease. J. Neurochem..

[53] Kitajima Y., Tashiro Y., Suzuki N., Warita H., Kato M., Tateyama M., Ando R., Izumi R., Yamazaki M., Abe M. (2014). Proteasome dysfunction induces muscle growth defects and protein aggregation. J. Cell Sci..

[54] Wang R., Zhao J., Zhang J., Liu W., Zhao M., Li J., Lv J., Li Y. (2015). Effect of lysosomal and ubiquitin-proteasome system dysfunction on the abnormal aggregation of alpha-synuclein in PC12 cells. Exp. Ther. Med..

[55] Kumar V., Singh D., Singh B.K., Singh S., Mittra N., Jha R.R., Patel D.K., Singh C. (2018). Alpha-synuclein aggregation, Ubiquitin proteasome system impairment, and L-Dopa response in zinc-induced Parkinsonism: Resemblance to sporadic Parkinson’s disease. Mol. Cell. Biochem..

[56] Silva G.M., Netto L.E.S., Simões V., Santos L.F.A., Gozzo F.C., Demasi M.A.A., Oliveira C.L.P., Bicev R.N., Klitzke C.F., Sogayar M.C. (2012). Redox Control of 20S Proteasome Gating. Antioxid. Redox Signal..

[57] Korovila I., Hugo M., Castro J.P., Weber D., Hohn A., Grune T., Jung T. (2017). Proteostasis, oxidative stress and aging. Redox Biol..

[58] Zmijewski J.W., Banerjee S., Abraham E. (2009). S-glutathionylation of the Rpn2 regulatory subunit inhibits 26 S proteasomal function. J. Biol. Chem..

[59] Demasi M., Shringarpure R., Davies K.J. (2001). Glutathiolation of the proteasome is enhanced by proteolytic inhibitors. Arch. Biochem. Biophys..

[60] Silva G.M., Netto L.E., Discola K.F., Piassa-Filho G.M., Pimenta D.C., Barcena J.A., Demasi M. (2008). Role of glutaredoxin 2 and cytosolic thioredoxins in cysteinyl-based redox modification of the 20S proteasome. FEBS J..

[61] Bridgford J.L., Xie S.C., Cobbold S.A., Pasaje C.F.A., Herrmann S., Yang T., Gillett D.L., Dick L.R., Ralph S.A., Dogovski C. (2018). Artemisinin kills malaria parasites by damaging proteins and inhibiting the proteasome. Nat. Commun..

[62] Nystrom T. (2005). Role of oxidative carbonylation in protein quality control and senescence. EMBO J..

[63] Grune T., Jung T., Merker K., Davies K.J. (2004). Decreased proteolysis caused by protein aggregates, inclusion bodies, plaques, lipofuscin, ceroid, and ‘aggresomes’ during oxidative stress, aging, and disease. Int. J. Biochem. Cell Biol..

[64] Cho M.-H., Cho K., Kang H.-J., Jeon E.-Y., Kim H.-S., Kwon H.-J., Kim H.-M., Kim D.-H., Yoon S.-Y. (2014). Autophagy in microglia degrades extracellular β-amyloid fibrils and regulates the NLRP3 inflammasome. Autophagy.

[65] Caccamo A., Ferreira E., Branca C., Oddo S. (2016). p62 improves AD-like pathology by increasing autophagy. Mol. Psychiatry.

[66] Cuervo A.M., Stefanis L., Fredenburg R., Lansbury P.T., Sulzer D. (2004). Impaired Degradation of Mutant α-Synuclein by Chaperone-Mediated Autophagy. Science.

[67] Ravikumar B., Duden R., Rubinsztein D.C. (2002). Aggregate-prone proteins with polyglutamine and polyalanine expansions are degraded by autophagy. Hum. Mol. Genet..

[68] Yung C., Sha D., Li L., Chin L.-S. (2016). Parkin Protects Against Misfolded SOD1 Toxicity by Promoting Its Aggresome Formation and Autophagic Clearance. Mol. Neurobiol..

[69] Bordi M., Berg M.J., Mohan P.S., Peterhoff C.M., Alldred M.J., Che S., Ginsberg S.D., Nixon R.A. (2016). Autophagy flux in CA1 neurons of Alzheimer hippocampus: Increased induction overburdens failing lysosomes to propel neuritic dystrophy. Autophagy.

[70] Nixon R.A., Yang D.-S. (2011). Autophagy failure in Alzheimer’s disease—Locating the primary defect. Neurobiol. Dis..

[71] Ramirez A., Heimbach A., Grundemann J., Stiller B., Hampshire D., Cid L.P., Goebel I., Mubaidin A.F., Wriekat A.L., Roeper J. (2006). Hereditary parkinsonism with dementia is caused by mutations in ATP13A2, encoding a lysosomal type 5 P-type ATPase. Nat. Genet..

[72] Zavodszky E., Seaman M.N., Moreau K., Jimenez-Sanchez M., Breusegem S.Y., Harbour M.E., Rubinsztein D.C. (2014). Mutation in VPS35 associated with Parkinson’s disease impairs WASH complex association and inhibits autophagy. Nat. Commun..

[73] Chen Y., Liu H., Guan Y., Wang Q., Zhou F., Jie L., Ju J., Pu L., Du H., Wang X. (2015). The altered autophagy mediated by TFEB in animal and cell models of amyotrophic lateral sclerosis. Am. J. Transl. Res..

[74] Levonen A.L., Hill B.G., Kansanen E., Zhang J., Darley-Usmar V.M. (2014). Redox regulation of antioxidants, autophagy, and the response to stress: Implications for electrophile therapeutics. Free Radic. Biol. Med..

[75] Scherz-Shouval R., Shvets E., Fass E., Shorer H., Gil L., Elazar Z. (2007). Reactive oxygen species are essential for autophagy and specifically regulate the activity of Atg4. EMBO J..

[76] Bensaad K., Cheung E.C., Vousden K.H. (2009). Modulation of intracellular ROS levels by TIGAR controls autophagy. EMBO J..

[77] Chen Y., Azad M.B., Gibson S.B. (2009). Superoxide is the major reactive oxygen species regulating autophagy. Cell Death Differ..

[78] Dodson M., Darley-Usmar V., Zhang J. (2013). Cellular metabolic and autophagic pathways: Traffic control by redox signaling. Free Radic. Biol. Med..

[79] Lee J., Giordano S., Zhang J. (2012). Autophagy, mitochondria and oxidative stress: Cross-talk and redox signalling. Biochem. J..

[80] Guerrero-Gomez D., Mora-Lorca J.A., Saenz-Narciso B., Naranjo-Galindo F.J., Munoz-Lobato F., Parrado-Fernandez C., Goikolea J., Cedazo-Minguez Á., Link C.D., Neri C. (2019). Loss of glutathione redox homeostasis impairs proteostasis by inhibiting autophagy-dependent protein degradation. Cell Death Differ..

[81] Civiletto G., Dogan S.A., Cerutti R., Fagiolari G., Moggio M., Lamperti C., Beninca C., Viscomi C., Zeviani M. (2018). Rapamycin rescues mitochondrial myopathy via coordinated activation of autophagy and lysosomal biogenesis. EMBO Mol. Med..

[82] Richardson A., Galvan V., Lin A.-L., Oddo S. (2015). How longevity research can lead to therapies for Alzheimer’s disease: The rapamycin story. Exp. Gerontol..

[83] Zhang L., Wang L., Wang R., Gao Y., Che H., Pan Y., Fu P. (2017). Evaluating the Effectiveness of GTM-1, Rapamycin, and Carbamazepine on Autophagy and Alzheimer Disease. Med. Sci. Monit..

[84] Thellung S., Scoti B., Corsaro A., Villa V., Nizzari M., Gagliani M.C., Porcile C., Russo C., Pagano A., Tacchetti C. (2018). Pharmacological activation of autophagy favors the clearing of intracellular aggregates of misfolded prion protein peptide to prevent neuronal death. Cell Death Dis..

[85] Choi D.H., Kim D.H., Park Y.G., Chun B.G., Choi S.H. (2002). Protective effects of rilmenidine and AGN 192403 on oxidative cytotoxicity and mitochondrial inhibitor-induced cytotoxicity in astrocytes. Free Radic. Biol. Med..

[86] Perera N.D., Sheean R.K., Lau C.L., Shin Y.S., Beart P.M., Horne M.K., Turner B.J. (2017). Rilmenidine promotes MTOR-independent autophagy in the mutant SOD1 mouse model of amyotrophic lateral sclerosis without slowing disease progression. Autophagy.

[87] Kim Y., Park J.K., Seo J.H., Ryu H.S., Lim K.S., Jeong M.H., Kang D.H., Kang S.W. (2018). A rapamycin derivative, biolimus, preferentially activates autophagy in vascular smooth muscle cells. Sci. Rep..

[88] Bossy-Wetzel E., Petrilli A., Knott A.B. (2008). Mutant huntingtin and mitochondrial dysfunction. Trends Neurosci..

[89] Gruber A., Hornburg D., Antonin M., Krahmer N., Collado J., Schaffer M., Zubaite G., Luchtenborg C., Sachsenheimer T., Brugger B. (2018). Molecular and structural architecture of polyQ aggregates in yeast. Proc. Natl. Acad. Sci. USA.

[90] Cenini G., Rub C., Bruderek M., Voos W. (2016). Amyloid beta-peptides interfere with mitochondrial preprotein import competence by a coaggregation process. Mol. Biol. Cell.

[91] Wang X., Becker K., Levine N., Zhang M., Lieberman A.P., Moore D.J., Ma J. (2019). Pathogenic alpha-synuclein aggregates preferentially bind to mitochondria and affect cellular respiration. Acta Neuropathol. Commun..

[92] Chaudhary R.K., Patel K.A., Patel M.K., Joshi R.H., Roy I. (2015). Inhibition of Aggregation of Mutant Huntingtin by Nucleic Acid Aptamers *In vitro* and in a Yeast Model of Huntington’s Disease. Mol. Ther..

[93] Patel K.A., Kolluri T., Jain S., Roy I. (2018). Designing Aptamers which Respond to Intracellular Oxidative Stress and Inhibit Aggregation of Mutant Huntingtin. Free Radic. Biol. Med..

[94] Mattson M.P., Goodman Y. (1995). Different amyloidogenic peptides share a similar mechanism of neurotoxicity involving reactive oxygen species and calcium. Brain Res..

[95] Lim Y.A., Rhein V., Baysang G., Meier F., Poljak A., Raftery M.J., Guilhaus M., Ittner L.M., Eckert A., Gotz J. (2010). Abeta and human amylin share a common toxicity pathway via mitochondrial dysfunction. Proteomics.

[96] Singh S., Bhowmick D.C., Pany S., Joe M., Zaghlula N., Jeremic A.M. (2018). Apoptosis signal regulating kinase-1 and NADPH oxidase mediate human amylin evoked redox stress and apoptosis in pancreatic beta-cells. Biochim. Biophys. Acta.

[97] Abramov A.Y., Duchen M.R. (2005). The role of an astrocytic NADPH oxidase in the neurotoxicity of amyloid beta peptides. Philos. Trans. R. Soc. B: Biol. Sci..

[98] Abeti R., Abramov A.Y., Duchen M.R. (2011). Beta-amyloid activates PARP causing astrocytic metabolic failure and neuronal death. Brain.

[99] Palazzi L., Bruzzone E., Bisello G., Leri M., Stefani M., Bucciantini M., Polverino de Laureto P. (2018). Oleuropein aglycone stabilizes the monomeric alpha-synuclein and favours the growth of non-toxic aggregates. Sci. Rep..

[100] Baruch-Suchodolsky R., Fischer B. (2008). Soluble amyloid beta1-28-copper(I)/copper(II)/Iron(II) complexes are potent antioxidants in cell-free systems. Biochemistry.

[101] Baruch-Suchodolsky R., Fischer B. (2009). Abeta40, either soluble or aggregated, is a remarkably potent antioxidant in cell-free oxidative systems. Biochemistry.

[102] Garzon-Rodriguez W., Yatsimirsky A.K., Glabe C.G. (1999). Binding of Zn(II), Cu(II), and Fe(II) ions to Alzheimer’s A beta peptide studied by fluorescence. Bioorganic Med. Chem. Lett..

[103] Khan A., Dobson J.P., Exley C. (2006). Redox cycling of iron by Abeta42. Free Radic. Biol. Med..

[104] Yang E.Y., Guo-Ross S.X., Bondy S.C. (1999). The stabilization of ferrous iron by a toxic beta-amyloid fragment and by an aluminum salt. Brain Res..

[105] Carija A., Navarro S., de Groot N.S., Ventura S. (2017). Protein aggregation into insoluble deposits protects from oxidative stress. Redox Biol..

[106] Sanchez de Groot N., Gomes R.A., Villar-Pique A., Babu M.M., Coelho A.V., Ventura S. (2015). Proteome response at the edge of protein aggregation. Open Biol..

[107] Sideri T.C., Stojanovski K., Tuite M.F., Grant C.M. (2010). Ribosome-associated peroxiredoxins suppress oxidative stress-induced de novo formation of the [PSI+] prion in yeast. Proc. Natl. Acad. Sci. USA.

[108] Cali T., Ottolini D., Negro A., Brini M. (2012). alpha-Synuclein controls mitochondrial calcium homeostasis by enhancing endoplasmic reticulum-mitochondria interactions. J. Biol. Chem..

[109] Guardia-Laguarta C., Area-Gomez E., Rub C., Liu Y., Magrane J., Becker D., Voos W., Schon E.A., Przedborski S. (2014). alpha-Synuclein is localized to mitochondria-associated ER membranes. J. Neurosci..

[110] Paillusson S., Gomez-Suaga P., Stoica R., Little D., Gissen P., Devine M.J., Noble W., Hanger D.P., Miller C.C.J. (2017). Alpha-Synuclein binds to the ER-mitochondria tethering protein VAPB to disrupt Ca(2+) homeostasis and mitochondrial ATP production. Acta Neuropathol..

[111] Pozo Devoto V.M., Dimopoulos N., Alloatti M., Pardi M.B., Saez T.M., Otero M.G., Cromberg L.E., Marín-Burgin A., Scassa M.E., Stokin G.B. (2017). αSynuclein control of mitochondrial homeostasis in human-derived neurons is disrupted by mutations associated with Parkinson’s disease. Sci. Rep..

[112] Tabner B.J., El-Agnaf O.M., German M.J., Fullwood N.J., Allsop D. (2005). Protein aggregation, metals and oxidative stress in neurodegenerative diseases. Biochem. Soc. Trans..

[113] Zhu C., Beck M.V., Griffith J.D., Deshmukh M., Dokholyan N.V. (2018). Large SOD1 aggregates, unlike trimeric SOD1, do not impact cell viability in a model of amyotrophic lateral sclerosis. Proc. Natl. Acad. Sci..

[114] Farrawell N.E., Lambert-Smith I., Mitchell K., McKenna J., McAlary L., Ciryam P., Vine K.L., Saunders D.N., Yerbury J.J. (2018). SOD1(A4V) aggregation alters ubiquitin homeostasis in a cell model of ALS. J. Cell Sci..

[115] D’Amico E., Factor-Litvak P., Santella R.M., Mitsumoto H. (2013). Clinical perspective on oxidative stress in sporadic amyotrophic lateral sclerosis. Free Radic. Biol. Med..

[116] Petrov D., Daura X., Zagrovic B. (2016). Effect of Oxidative Damage on the Stability and Dimerization of Superoxide Dismutase 1. Biophys. J..

[117] Dedeoglu A., Cormier K., Payton S., Tseitlin K.A., Kremsky J.N., Lai L., Li X., Moir R.D., Tanzi R.E., Bush A.I. (2004). Preliminary studies of a novel bifunctional metal chelator targeting Alzheimer’s amyloidogenesis. Exp. Gerontol..

[118] Sharma A.K., Pavlova S.T., Kim J., Finkelstein D., Hawco N.J., Rath N.P., Kim J., Mirica L.M. (2012). Bifunctional Compounds for Controlling Metal-Mediated Aggregation of the Aβ42 Peptide. J. Am. Chem. Soc..

[119] Rajasekhar K., Mehta K., Govindaraju T. (2018). Hybrid Multifunctional Modulators Inhibit Multifaceted Abeta Toxicity and Prevent Mitochondrial Damage. ACS Chem. Neurosci..

[120] Hilt S., Altman R., Kalai T., Maezawa I., Gong Q., Wachsmann-Hogiu S., Jin L.W., Voss J.C. (2018). A Bifunctional Anti-Amyloid Blocks Oxidative Stress and the Accumulation of Intraneuronal Amyloid-Beta. Molecules.

